# Camera-Based, Non-Contact, Vital-Signs Monitoring Technology May Provide a Way for the Early Prevention of SIDS in Infants

**DOI:** 10.3389/fneur.2016.00236

**Published:** 2016-12-23

**Authors:** Fang Zhao, Meng Li, Zhongyi Jiang, Joe Z. Tsien, Zhaohui Lu

**Affiliations:** ^1^Brain and Behavior Discovery Institute and Department of Neurology, Medical College of Georgia, Augusta University, Augusta, GA, USA; ^2^Banna Biomedical Research Institute, Xi-Shuang-Ban-Na, Yunnan, China; ^3^Department of Thoracic and Cardiac Surgery, Shanghai Children’s Medical Center, Affiliated with Shanghai Jiaotong University School of Medicine, Shanghai, China

**Keywords:** SIDS, prevention, non-contact monitoring, vital signs, camera, video

## Abstract

Sudden infant death syndrome (SIDS) is the unexplained death, usually during sleep, of a baby younger than 1-year-old. Even though researchers have discovered some factors that may put babies at extra risk, SIDS remains unpredictable up until now. One hypothesis is that impaired cardiovascular control may play a role in the underlying mechanism of SIDS. A reduction of heart rate variability (HRV) and progressive decrease in heart rate (HR) have been observed in infants who have later succumbed to SIDS. Many clues indicated the heart could be the final weakness in SIDS. Therefore, continuous monitoring of the dynamic changes within the heart may provide a possible preventive strategy of SIDS. Camera-based photoplethysmography was recently demonstrated as a contactless method to determine HR and HRV. This perspective presents a hypothesis that a camera-based, non-contact, vital-sign monitoring technology, which can indicate abnormal changes or a sudden loss of vital signs in a timely manner, may enable a crucial and low-cost means for the early prevention of SIDS in newborn infants.

## Impaired Cardiovascular Control and Sudden Infant Death Syndrome (SIDS)

Sudden infant death syndrome, which usually occurs during sleep with a peak incidence at 2–3 months of age, remains as one of the current leading causes of post-neonatal infant death. The pathophysiological mechanism underlying the death is still unclear. This old problem will continue to plague us until the answers eventually fall into place. The majority of findings suggests that impaired cardiovascular control could lead to SIDS. Infants who have succumbed to SIDS have a higher basal heart rate (HR) (1), reduced heart rate variability (HRV) (2, 3), familial long QT syndrome (4), Brugada syndrome (5), lower parasympathetic activity, and/or higher sympathovagal balance (6).

There are many clinical studies that have linked a progressive decrease in HR with SIDS cases (7–13). These studies are based on the objective data that immediately precede SIDS obtained from physiological memory-monitor recordings. The primary event in every case was a progressive decrease in HR that developed over minutes or hours before SIDS (14). The exact causes of the bradycardia could not be determined from the recordings, but there is an assumption that the progressive decrease in HR is probably due to hypoxic cardiac depression, which was deduced from the evidence obtained from similar recordings during apparent life-threatening events (ALTEs).

Subsequently, some risk factors related to autonomic dysfunction have been implicated in SIDS. Reduction in cardiac autonomic function induced by these risk factors has been demonstrated by the analysis of the spontaneous beat-to-beat changes in HR, known as HRV. Prone sleeping is well established as a major risk factor for SIDS (15), which is associated with a reduction in cardiovascular and autonomic control (16–18). HRV parameters were found to be reduced in prone position compared to supine position, suggesting diminished sympathetic activity in prone position (17–20). Maternal cigarette smoking is another modifiable risk factor for SIDS. The increased risk for SIDS associated with maternal smoking has been attributed to various mechanisms, including impairment of autonomic functions (21, 22). Infants from smoking mothers have reduced HRV and lower frequency power normalized to the total spectral power (LF/TP) (23). Larger infant HR decreases during hyperoxia and smaller HR rises during hypoxia are correlated with the increasing number of cigarettes smoked by the mother (24). Thermal stress could also increase an infant’s vulnerability to SIDS by disrupting autonomic functions (25). The elevated ambient temperature is associated with a higher basal HR and lower short- and long-term HRV during all sleep stages (26), and a cool environment is linked to greater autonomic dysfunction (27). It has been suggested that immature autonomic cardiovascular control contributes to the increase risk for SIDS in preterm infants. The delayed maturation of autonomic cardiovascular control in preterm infants, reflected in a shorter inter-beat (RR)-interval (28) and a lower power of HRV (29, 30).

The high incidence, unknown pathophysiological mechanisms, and the catastrophic impact on affected families are what make SIDS so frightening. Given the strong and mounting evidence implicating reduced cardiovascular control in SIDS cases, questions regarding home monitoring in order to provide the warning needed in time for intervention arise in families.

## Limitations on Current HR Monitoring Techniques

Current HR monitoring techniques usually require the use of adhesive electrodes or sensors. Long-term attachment to the sensors can cause skin irritation, stress, pain, and even damage to the fragile skin of infants. In particular, epidermal stripping from removal of adhesives may occur, particularly in preterm infants less than 27 weeks of gestational age due to incompletely developed stratum corneum and the decreased number of fibrils that connect the epidermis to the dermis (31, 32). Moreover, the obtrusiveness of the wires has a negative impact on parent-child bonding, especially during the care by a kangaroo mother. The stress caused by the repetitive application and removal of patches can interfere with the infant’s normal growth and hamper the infant’s cognitive development (33, 34).

An alternative solution might be technologies for non-contact monitoring like capacitive ECG (35), ballistocardiograph (36), Laser Doppler (37), Microwave Doppler Radar (38), Ultra-Wideband Radar (39, 40), or thermography (41). In spite of varying success that these technologies have obtained, they share some common problems in that they all require expensive and specialized hardware. One of the most recent and promising non-contact techniques is the camera-based photoplethysmography (PPG) of sensing the blood volume pulse (BVP) through variations in reflected light (42). Its ease of use, low cost, and convenience make it a tantalizing prospect that would enhance the delivery of primary health care for infants.

## Camera-Based, Non-Contact, Vital-Signs Monitoring Technologies

There have been many attempts made to develop an ambient light-based approach, utilizing normal ambient light as the source of light (43–51). The ability to accurately measure cardiovascular parameters – namely, HR and HRV—*via* a simple consumer-level digital camera with normal ambient light as the light source has been demonstrated on healthy adult humans in controlled environments for short periods of time. Many ambient light-based approaches are based on the fact that the red, green, and blue (RGB) channels that are produced by ambient light register the BVP with different relative strengths (46–51). The underlying BVP can be the recovery from a linear combination of RGB channels. Poh et al. exploited this difference to achieve motion robustness by recovering independent source signals from a linear combination of RGB channels using ICA (46, 47). The linear combination coefficients could be estimated by maximizing the non-Gaussianity within ICA output components. It has been demonstrated that utilizing ICA could further attenuate the motion artifact and improve the estimation accuracy of BVP signals, when compared with using the raw green channel without ICA. Lewandowska and Nowak later utilized PCA instead of ICA to discover the linear combinations of RGB channels (48). De Haan and Jeanne proposed a chrominance-based approach that constructs two orthogonal chrominance signals from the linear combination of RGB color channels (49). It is superior in the SNR in the presence of periodic motion artifacts. More recently, McDuff et al. moved a step forward through employing a novel five-band camera with red, green, blue, cyan, and orange color channels (51), which provides higher flexibility in the number of source signals for blind source separation methods, like PCA and ICA. The authors showed that the orange channel helps boost the measurement performance and the GCO channel combination outperforms the RGB channels showing the best performance in HR and HRV measurement. Regarding distance, a measureable distance up to 3 m was observed with GCO channel combination.

There are also some papers in the literature that reported a camera-based estimation of HR in the neonatal intensive care unit (NICU) with the normal NICU lighting as the illumination source (52–55). Scalise et al. demonstrated for the first time the feasibility of camera-based PPG for the NICU on seven infants (52). An algorithm based on ICA was used in this study to measure HR on a 30-s video with a webcam 20 cm away from the face. Aarts et al. studied 19 infants in the NICU in both the USA and the Netherlands (53). Recordings were up to 5 min with the camera at approximately 1 m from the infants. A good result, which is defined as a HR obtained from fast Fourier transform (FFT) of the green channel signal matching control in >90% of the time, has derived in 13 out of 19 infants. Villarroel et al. continuously recorded two preterm infants using a digital video camera for nearly a total of 40 h in the high-dependency care area of the NICU in Oxford (54). The authors have shown that continuous estimating of HR, RR, and changes in SpO_2_ with an accuracy that is clinically useful can be achieved with their chrominance-based algorithms (50). Episodes of bradycardia (decrease of HR) accompanied by a major desaturation, as a result of the immaturity of the cardiorespiratory system, can be identified. The Xerox Innovation Group – PARC monitored eight neonates with gestational ages of 37–40 weeks in the NICU (55). Video data from the infants’ chests and faces were recorded for 30 min each, with a camera positioned at a distance of ~3 feet. Mean bias of 2.52 bpm and 95% limits of agreement of ±5.48 bpm, which are close to medical standards, were obtained.

**Figure 1:**
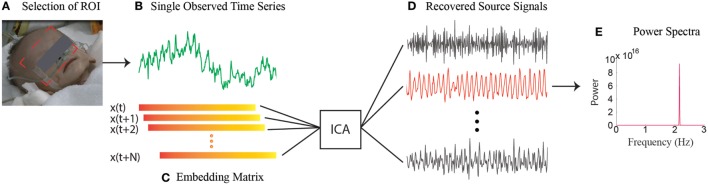
**Overview of the single-channel based approach**. **(A)** Selection of the ROI. **(B)** Spatial average of pixels in ROI over time yields a raw single-channel signal. **(C)** Construction of the embedding matrix by a number of consecutive delay vectors from the raw single-channel signal. **(D)** Recovery of source signals by implementing ICA on embedding matrix. **(E)** The power spectra of the selected source signal.

Regardless of how successful these ambient-light-based methods have been in acquiring physiological parameters, a key limitation regarding the utilization of ambient light as the illumination source is that they cannot work when not enough ambient light is available, such as during nighttime. However, the ability to function in low light or without visible light is particularly intriguing for the study of SIDS since the SIDS events happen exclusively during sleep and often in dimly lit rooms. To conquer this limitation, we have developed a single-channel based algorithm (56) that can isolate multiple underlying components using only the temporal information inherent in the single-channel recordings. Therefore, our method lends itself well to applications where multichannel images are not available or are undesirable, such as during nighttime. Figure 1 provides an overview of our approach. At the heart of our method is to perform ICA on the embedding matrix that is constructed out of a series of delay vectors from the single-channel recordings. In our initial study, we demonstrated non-contact measurements of HR for both day and night conditions on 15 adults and a 1-month-old infant while the baby is asleep (56). We mimicked the night in a dark room with a near-infrared LED serving as a source of illumination. We have now begun a clinical study with infants at Shanghai Children’s Medical Center in China. Our single-channel method that has the potential to automatically detect the onset of devastating events, such as SIDS, at night and promptly alert parents, family members, or medical staff will be of significant value to patients and family. It can also improve efficiency and reduce medical and legal costs for hospitals and society.

## Conclusion

There is a lack of study that could provide evidence as to whether or not home monitoring could provide warning in time for intervention—or even if intervention would prevent the unexpected death. One of the unanswered questions regarding home monitoring is how to identify the infants who may be at risk for SIDS to be monitored. Evidence that siblings of SIDS victims and infants who have had episodes of extreme apnea, bradycardia, or an ALTE are at any increased risk for SIDS is inconclusive and inadequate. Furthermore, false alarms can occur when the sensors inadvertently are shifted out of position due to infant’s moving in the bed. Frequent false alarms may increase parents’ depression and hostility. Moreover, widespread adoption of home monitoring has been limited by the cost associated with purchasing the device and the low utilization coefficient by the parents (who may unwilling to attach the special designed sensors on their baby). With the emergence of camera-based, non-contact technologies, the camera integrated in mobile phones, tablets, or notebooks could easily double as a heart-rate monitor. In particular, the HR measurement function could also be readily integrated in infant or home-security monitoring systems. The non-contact nature makes the camera-based technology an ideal mean for HR and HRV monitoring for infants. Given the evidence that a reduction of HRV and progressive decrease in HR has been observed in infants who have later succumbed to SIDS, a camera-based, non-contact vital-sign monitoring technology, which can indicate abnormal changes or a sudden loss of vital signs in a timely manner, may enable a crucial and low-cost means for the early prevention of SIDS in newborn infants.

## Author Contributions

FZ, ZJ, and JZT conceived the idea. FZ, ML, and ZL wrote the paper.

## Conflict of Interest Statement

The authors declare that the research was conducted in the absence of any commercial or financial relationships that could be construed as a potential conflict of interest.
